# Transglycosylation products generated by *Talaromyces amestolkia*e GH3 β-glucosidases: effect of hydroxytyrosol, vanillin and its glucosides on breast cancer cells

**DOI:** 10.1186/s12934-019-1147-4

**Published:** 2019-05-31

**Authors:** Juan Antonio Méndez-Líter, Isabel Tundidor, Manuel Nieto-Domínguez, Beatriz Fernández de Toro, Andrés González Santana, Laura Isabel de Eugenio, Alicia Prieto, Juan Luis Asensio, Francisco Javier Cañada, Cristina Sánchez, María Jesús Martínez

**Affiliations:** 1 Department of Microbial and Plant Biotechnology, Centro de Investigaciones Biológicas, CSIC, Ramiro de Maeztu 9, 28040 Madrid, Spain; 2 Department of Biochemistry and Molecular Biology, Complutense University, Madrid, Spain; 3 Instituto de Investigación Hospital 12 de Octubre, Madrid, Spain; 4 Department of Chemical and Physical Biology, Centro de Investigaciones Biológicas, CSIC, Ramiro de Maeztu 9, 28040 Madrid, Spain; 5 Glycochemistry and Molecular Recognition Group, Instituto de Química Orgánica General (IQOG-CSIC), Calle Juan de la Cierva, 3, 28006 Madrid, Spain

**Keywords:** Transglycosylation, β-Glucosidases, Glycosyl hydrolases, Hydroxytyrosol, Vanillin, Glucosides, Breast cancer cells

## Abstract

**Background:**

Transglycosylation represents one of the most promising approaches for obtaining novel glycosides, and plant phenols and polyphenols are emerging as one of the best targets for creating new molecules with enhanced capacities. These compounds can be found in diet and exhibit a wide range of bioactivities, such as antioxidant, antihypertensive, antitumor, neuroprotective and anti-inflammatory, and the eco-friendly synthesis of glycosides from these molecules can be a suitable alternative for increasing their health benefits.

**Results:**

Transglycosylation experiments were carried out using different GH3 β-glucosidases from the fungus *Talaromyces amestolkiae*. After a first screening with a wide variety of potential transglycosylation acceptors, mono-glucosylated derivatives of hydroxytyrosol, vanillin alcohol, 4-hydroxybenzyl alcohol, and hydroquinone were detected. The reaction products were analyzed by thin-layer chromatography, high-pressure liquid chromatography, and mass spectrometry. Hydroxytyrosol and vanillyl alcohol were selected as the best options for transglycosylation optimization, with a final conversion yield of 13.8 and 19% of hydroxytyrosol and vanillin glucosides, respectively. NMR analysis confirmed the structures of these compounds. The evaluation of the biological effect of these glucosides using models of breast cancer cells, showed an enhancement in the anti-proliferative capacity of the vanillin derivative, and an improved safety profile of both glucosides.

**Conclusions:**

GH3 β-glucosidases from *T. amestolkiae* expressed in *P. pastoris* were able to transglycosylate a wide variety of acceptors. Between them, phenolic molecules like hydroxytyrosol, vanillin alcohol, 4-hydroxybenzyl alcohol, and hydroquinone were the most suitable for its interesting biological properties. The glycosides of hydroxytyrosol and vanillin were tested, and they improved the biological activities of the original aglycons on breast cancer cells.

**Electronic supplementary material:**

The online version of this article (10.1186/s12934-019-1147-4) contains supplementary material, which is available to authorized users.

## Background

Glycosyl hydrolases (GHs) are enzymes that hydrolyze glycosidic linkages, and are essential in nature for the multiplicity of roles that they play. The huge diversity of natural carbohydrates is directly correlated with the wide variety of GH-type activities reported so far [1]. The traditional concept of glycosidases mostly refers to their hydrolytic capacities, and their current applications are mainly associated to the degradation of lignocellulosic biomass and food industry [2]. Retaining glycosyl hydrolases act through a double-displacement mechanism involving the formation of a covalent glycosyl-enzyme intermediate, which is subsequently cleaved upon nucleophilic attack by water (hydrolysis reactions). However, they can also produce new glycosidic bonds when alternative nucleophiles to water participate as acceptors, through a mechanism named transglycosylation [3]. As a result of the latter reaction, a new glycoside is synthesized by the transfer of a sugar unit (e.g.: glucose, galactose, xylose, fructose…) to the nucleophilic acceptor.

The ability of GHs to catalyze transglycosylation reactions has made these enzymes a suitable alternative to chemical approaches for obtaining different glycosides [4]. These enzymatic conversions allow a more environmentally friendly synthesis of these compounds, compared to the current chemical approaches, which usually imply the formation of toxic byproducts, need more steps, and display lower regio- and stereoselectivity [5]. On the other hand, glycosidases outstand for having a complete stereoselectivity and a remarkably greater regioselectivity [5].

Many beneficial effects of glycosides have been reported, for instance: increasing solubility of the original compound [6–8], making the new compound safer [9] or improving its stability [8, 10, 11]. In general, enzymatic transglycosylation by glycosidases is considered as a good and eco-friendly alternative over traditional chemical synthesis for obtaining novel molecules with added value.

Phenolic compounds are molecules that possess one or more aromatic rings with one or more hydroxyl groups. They are broadly distributed in the Plant Kingdom, and are the most abundant secondary metabolites in plants, ranging from simple molecules such as aromatic acids, to highly polymerized substances like tannins [12]. Recently, the health effects of many phenolic compounds have come to the attention of nutritionists, mainly due to their antioxidant capacity and their potential effects in the prevention of various diseases associated to oxidative stress. The demonstrated effects of these plant metabolites, in terms of cardiovascular, neurodegenerative diseases and cancer [13], have postulated them as an interesting option for preventing or even treating diseases. Nevertheless, one of the main disadvantages of these molecules is their usually poor bioavailability when administered to humans.

*Talaromyces amestolkiae* has been postulated as a great GH producer [14, 15] whose highly efficient β-glucosidases (BGLs) from the family GH3 deserve special attention. In this context, BGL-2 was the first fully characterized BGL that possessed a cellulose-binding domain (CBD), and it displayed a very high efficiency against cellobiose and other oligosaccharides. A truncated version, without the CBD region (BGL-2T) keeps the efficiency in hydrolytic reactions [16]. The other GH3 β-glucosidase characterized from this fungus, named BGL-3, is a versatile enzyme produced under carbon starvation, that hydrolyzes efficiently typical 1,4-β-glucosidase substrates, but also shows high activity over 1,3-β-glucose bonds [17].

The aim of this work is to study the transglycosylation profile of these β-glucosidases (BGL-2, BGL-2T and BGL-3) using different acceptors, and to test the properties of the most interesting glycosides as potential antitumor agents in preclinical models of cancer.

## Results and discussion

### The BGLs of *T. amestolkiae* are versatile tools for transglycosylation

The cellulolytic system of *T. amestolkiae* has shown an outstanding hydrolytic efficiency over cellulose, but the potential of some of these enzymes in the synthesis of novel compounds by transglycosylation has not been evaluated so far. Many GHs can catalyze this kind of reaction, and numerous strategies have been developed in order to obtain added-value compounds by transglycosylation [18–23]. In order to test the transglycosylation activity of the GH3 β-glucosidases BGL-2, BGL-2T and BGL-3, we first performed a screening of potential acceptors, according to the methodology previously developed [24]. The 70 compounds tested (listed in Table 1) encompass a wide variety of alcohols including sugars, sterols, phenolic compounds, or amino acids. It is very important to remark that this method allows analyzing a great number of compounds, but the most interesting positive hits identified for each enzyme in this screening should be subjected to a second screening by Thin Layer chromatography to corroborate the results, thus discarding false positives. Out of the 70 potential acceptors assayed, 32, 31 and 35 were determined as positive hits for BGL-2, BGL-2T and BGL-3, respectively (Table 1). This number of positive hits for each BGL was high compared to those previously reported for other GHs [24], but relatively low if compared to what has been recently published for a β-xylosidase from *T. amestolkiae* [21]: It is remarkable that the three assayed enzymes were able to produce glucosides of several phenolic compounds, many of them with reported antioxidant properties, such as epigallocatechin gallate (EGCG), hydroxytyrosol, vanillyl alcohol, 4-hydroxybenzyl alcohol, or hydroquinone. However most of them have a poor bioavailability, which limits their use in many industrial or biomedical applications. In this regard, their glycosylation may provide a solution to this problem by converting them into more soluble conjugates. Other positive hits for the three enzymes were detected when 4-nitrophenol galacto-, gluco- and xylo-pyranosides, and also disaccharides like lactose or melibiose, were used as acceptors. These results open up the possibility for synthesizing different oligosaccharides in a regio- and stereoselective manner. Many of these molecules have shown a great prebiotic effect, which is related with health benefits [25, 26]. In addition, the synthesis of disaccharide derivatives of 4-nitrophenol seems to be promising, since they could be used as novel substrates for other GHs.

**Table 1:** Inhibition recovery of *T. amestolkiae* BGLs in the presence of different molecules

Molecule	BGL-2	BGL-2T	BGL-3
1-Heptanol	–	–	+
1-Propanol	+	–	–
2,4-Dinitrophenol	–	–	–
2,6-Dihydroxynaphthalene	–	–	+ +
2-Butanol	–	+	–
2-Mercaptoethanol	–	+ + +	+
*2*-Nitrophenyl β-D-glucopyranoside	–	–	–
2-Propanol	+ + +	+	+
3,3-Diphenyl propanol	–	+	+ +
4-Cresol	+	+ +	–
4-Hydroxybenzyl alcohol	+	+ + +	+
4-Methylumbilliferyl β-D-xylopyranoside	+ + +	+ +	
4-Nitrophenol	–	–	+ + +
*4*-Nitrophenyl α-arabinopyranoside	–	+	–
*4*-Nitrophenyl α-D-glucopyranoside	+	+ +	+
*4*-Nitrophenyl α-D-rhamnopyranoside	–	–	+ +
4-Nitrophenyl β-D-fucopyranoside	–	–	–
*4*-Nitrophenyl β-D-galactopyranoside	+ +	+ + +	+ + +
*4*-Nitrophenyl β-D-glucopyranoside	+ + +	+ + +	+ + +
*4*-Nitrophenyl β-D-xylopyranoside	+ + +	+ + +	+ + +
L-Arabinose	–	–	–
Arabitol	–	+ + +	–
Ascorbic acid	–	–	–
Catechol	–	+	+ +
Cellobiose	–	–	–
Cinnamyl alcohol	+	–	+ + +
Cyclohexanol	–	–	+
Dulcitol	+ + +	+	+ + +
EGCG	+ + +	+ + +	+ + +
Ergosterol	+ + +	+	+
Ethanol	–	+	–
Eugenol	–	–	+ + +
Ferulic acid	–	–	–
d-Fructose	–	–	–
D-Galactose	+	+	–
Gallic acid	+ +	–	–
Gentiobiose	–	–	–
d-Glucose	–	–	–
Glycerol	+	–	+ + +
Guaiacol	–	–	–
Hydroquinone	+	+	+
Hydroxytyrosol	+ + +	+ + +	+ + +
*myo*-Inositol	+ + +	+ + +	+ + +
Lactose	+ + +	+ + +	+ + +
Maltose	–	+ +	+ + +
Mannitol	+ +	+ + +	–
d-Mannose	–	–	–
Melibiose	+	+	+ +
Menthol	–	–	+
Methanol	+	–	–
Naphthol	+	+	+
Phenol	+ + +	+ + +	+
Propargyl alcohol	–	–	–
Quercetin	–	–	–
Raffinose	+	+	+ + +
Resveratrol	+	–	–
D-Ribose	–	–	–
l-Serine	–	+ +	+ + +
Sorbitol	+ + +	–	+ +
Sorbose	–	–	–
Sucrose	–	+	–
l-Threonine	–	–	+
L-Trehalose	+	–	–
l-Tyrosine	–	–	+ + +
Vanillyl alcohol	+	+ +	+
Xylitol	+ + +	–	–
D-Xylose	–	–	–
α-Tocopherol	–	–	–
β-Sitosterol	–	–	–

The hits with higher recovered activity than no-acceptor control were considered potential acceptors of transglycosylation. In the table, acceptor efficiency was defined by symbols, being +++ which represents the best efficiency, and – if there is no activity recovered

In the case of BGL-3, the glycosylation of l-serine, l-threonine, and l-tyrosine is very remarkable. Protein glycosylation is considered of fundamental importance [27], thus the formation of glycosidic bonds with amino acids may have many applications, from serving as an assembly for synthetic glycopeptides that can be used for triggering tumor-cell-specific immune response, to acting as ligands of carbohydrate-binding proteins or as enzyme substrates or inhibitors [28].

It is also worth noting that, although the three assayed BGLs belong to the same glycosyl hydrolase family, some differences can be found among their positive acceptor profiles (Table 1). This diversity in the transglycosylation profiles, even among enzymes that are considerably similar in terms of sequence, generates expectations of wider but selective applications via different enzyme variants. These variations could seem surprising among BGL-2 forms, although they agree with the different hydrolytic efficiencies over *p*-nitrophenol sugars and cellooligosaccharides shown by BGL-2 and BGL-2T, two enzymes that only differ on the presence or absence of the CBD.

### Screening of transglycosylation products by TLC

**Fig. 1:**
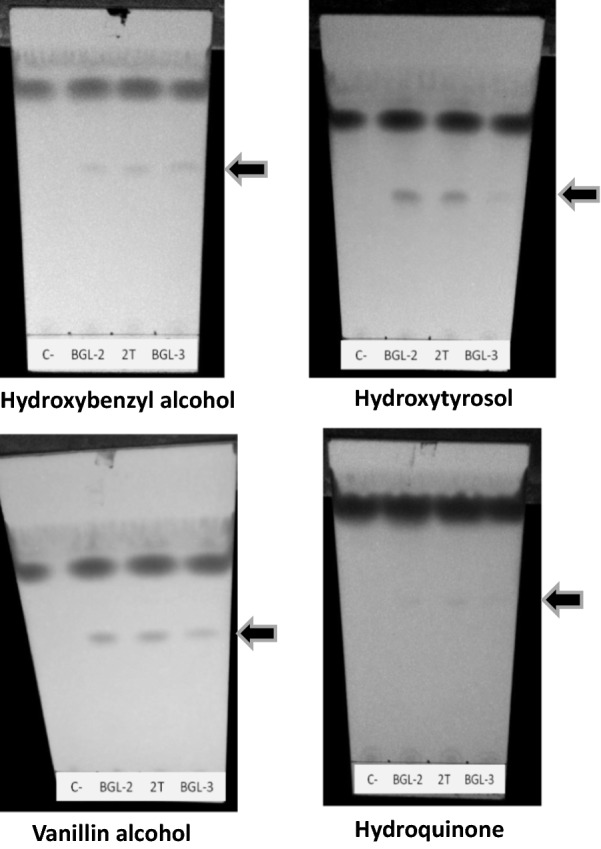
Thin layer chromatography analysis of transglycosylation reactions of hydroxytyrosol, vanillyl alcohol, hydroxybenzyl alcohol and hydroquinone. Arrows point at the reaction products

After the first screening, several phenolic antioxidants with potential biotechnological applications and solubility limitations that could be avoided by glycosylation were submitted to a second screening to eliminate false positive acceptors. These compounds were EGCG, hydroxytyrosol, hydroquinone, 4-hydroxybenzyl alcohol, and vanillyl alcohol. Non identifiable glycosylated derivatives were detected for EGCG, which discarded it as an effective transglycosylation acceptor in this system, in spite of the positive results of the preliminary screening, which ratifies the necessity of performing a second assay to confirm BGL acceptors. However, the other phenolic compounds evaluated did show their corresponding glucoside bands on TLC (Fig. 1).

### HPLC and MS analysis of the transglycosylation products and selection of the most efficient BGL

**Fig. 2:**
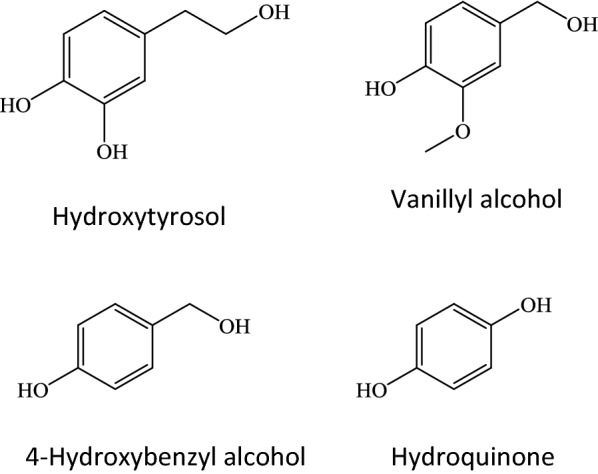
Molecular structures of the positive acceptor hits of transglycosylation

In order to determine which of the evaluated BGLs displayed a higher yield in glucoside synthesis, the transglycosylation products of hydroxytyrosol, hydroquinone, 4-hydroxybenzyl alcohol and vanillyl alcohol (Fig. 2) were analyzed and quantified by HPLC. The peaks from these glucosides were detected by their absorbance at 270 nm, since all of the selected compounds contain aromatic rings and, at this wavelength, the remaining cellobiose or other side products, such as glucose, did not interfere with the analysis. The results (Table 2) showed that yields obtained with BGL-2 and BGL-2T were very similar in every case, whereas transglycosylation yield catalyzed with BGL-3 was lower. In addition, these analyses corroborated the higher efficiency as acceptors of hydroxytyrosol, 4-hydroxybenzyl alcohol and vanillyl alcohol over hydroquinone, since the corresponding product yield was five- to tenfold higher.

**Table 2:** Comparison of the transglycosylation activity of BGLs from *T. amestolkiae*

Glycosylated product	BGL-2	BGL-2T	BGL-3
Hydroxytyrosol	2.41	2.56	0.89
Vanillyl alcohol	4.09	3.65	1.72
Hydroquinone	0.35	0.42	0.28
4-hydroxybenzyl alcohol	1.63	1.54	0.83

Final product yields are given in mM concentration

Since BGL-2 was produced with a better yield and was easier to purify than BGL-2T [16], this enzyme was selected for scaling up the transglycosylation reaction for the four selected glucosides. The presence of the expected mono-glucosylated derivative for each acceptor was corroborated by ESI–MS (Table 3).

**Table 3:** ESI-MS data for the products obtained by transglycosylation catalyzed by BGL-2

Glycoside	Intensity	m/z
Hydroxytyrosol	40,201	339.0
Vanillyl alcohol	240,349	339.0
4-Hydroxybenzyl alcohol	110,093	309.0
Hydroquinone	26,153	295.0

All the glycosides were detected as Na^+^ adducts

All the synthesized products are potentially interesting from a biotechnological point of view, since their phenolic precursors already outstand for their applications. Several studies have remarked that hydroxytyrosol has antioxidant, anti-proliferative and anti-inflammatory activities, and beneficial effects on the cardiovascular system by preventing oxidative stress [22, 29]. Hydroquinone is an interesting molecule as a water-soluble reducing agent, but its main applications are related to skin depigmentation, although its side effects on human health are an increasing concern [30]. With this idea in mind, an approximation from a prodrug point of view could be really interesting, with the hydroquinone being potentially inactivated in its glycosylated form, and then activated after the action of endogenous glycosidases. Although vanillyl alcohol has been scarcely used, vanillin has a wide variety of applications as a flavoring agent in food or beverages [31], but also for having potential anti-proliferative and neuroprotective effects [32, 33]. The anti-angiogenic, anti-inflammatory and anti-nociceptive applications of 4-hydroxybenzyl alcohol make it one of the best-known phenolic compounds isolated from plants [34, 35]. Out of the four products synthesized by transglycosylation with BGL-2, and on the basis of the interesting properties of their corresponding precursors and the good transglycosylation yields obtained in previous experiments, the glucosides of hydroxytyrosol and vanillyl alcohol were selected to optimize their production and to test their biological activity.

### Optimization of glucosides synthesis by response surface methodology

The reaction conditions for hydroxytyrosol and vanillyl alcohol glucosides were optimized using a response surface method, the Box–Behnken design, in order to improve their production. The matrix of experiments generated comprised 50 reactions for each compound.

The results were analyzed using the Design-Expert^®^ software. The optimum conditions for producing each transglycosylation product were determined by quadratic equations and can be seen in Additional file 1: Data S1. Both models predicted the glucoside production as a function of the concentrations of hydroxytyrosol and vanillyl alcohol, the concentration of cellobiose, the enzyme units, the temperature and the time of reaction. The analysis of variance test performed by the software validated the experimental data.

The Design Expert software allows selecting “maximum production yield” or “maximum conversion rate” as the parameters for optimization of the experimental conditions. As a general rule, the highest productions were obtained when donor and acceptor are added at their maximum concentrations, as it has been reported previously for other β-glucosidases [36].

However, maximum conversion rates relative to initial acceptor concentrations were only reached when the donor was added at the maximum concentration tested and the acceptor was in low amounts.

The conditions for maximum production generated by the software required the same reaction mixtures for each compound: 4 U of β-glucosidase activity of BGL-2, 350 mM of cellobiose as donor, 195 mM of hydroxytyrosol or vanillyl alcohol as acceptors, 50 mM acetate pH 4 and 0.1% BSA. They were incubated at 50 °C for 5 h. However, for maximum relative conversion of the acceptors, a concentration of hydroxytyrosol and vanillyl alcohol of 32 mM was used, maintaining the above settings for the remaining variables. We obtained a “maximum production yield” value of 2.55 g/L (8 mM) for hydroxytyrosol glucoside, and 3.8 g/L (12 mM) for vanillyl glucoside, and “maximum conversion rate” of 13.8% and 19% for hydroxytyrosol and the vanillyl alcohol glucosides, respectively. These glucosides were purified by HPLC, as described in materials and methods prior to their characterization by NMR.

### Solubility of new glycosides and characterization by NMR

The aqueous solubility of each glycoside at room temperature was compared with that of the respective aglycon. Solubility detected for hydroxytyrosol was of 88.84 mg/mL, and the glycoside had its solubility increased up to 254.75 mg/mL. On the other hand, vanillyl alcohol showed a solubility of 16.12 mg/mL, which was improved by transglycosylation to a value of 165.32 mg/mL. This data confirmed the improvement of the solubility of the novel molecules, making them more bio-available. A similar effect were detected recently in a work that developed the α-glycosylation of pterostilbene [37].

**Fig. 3:**
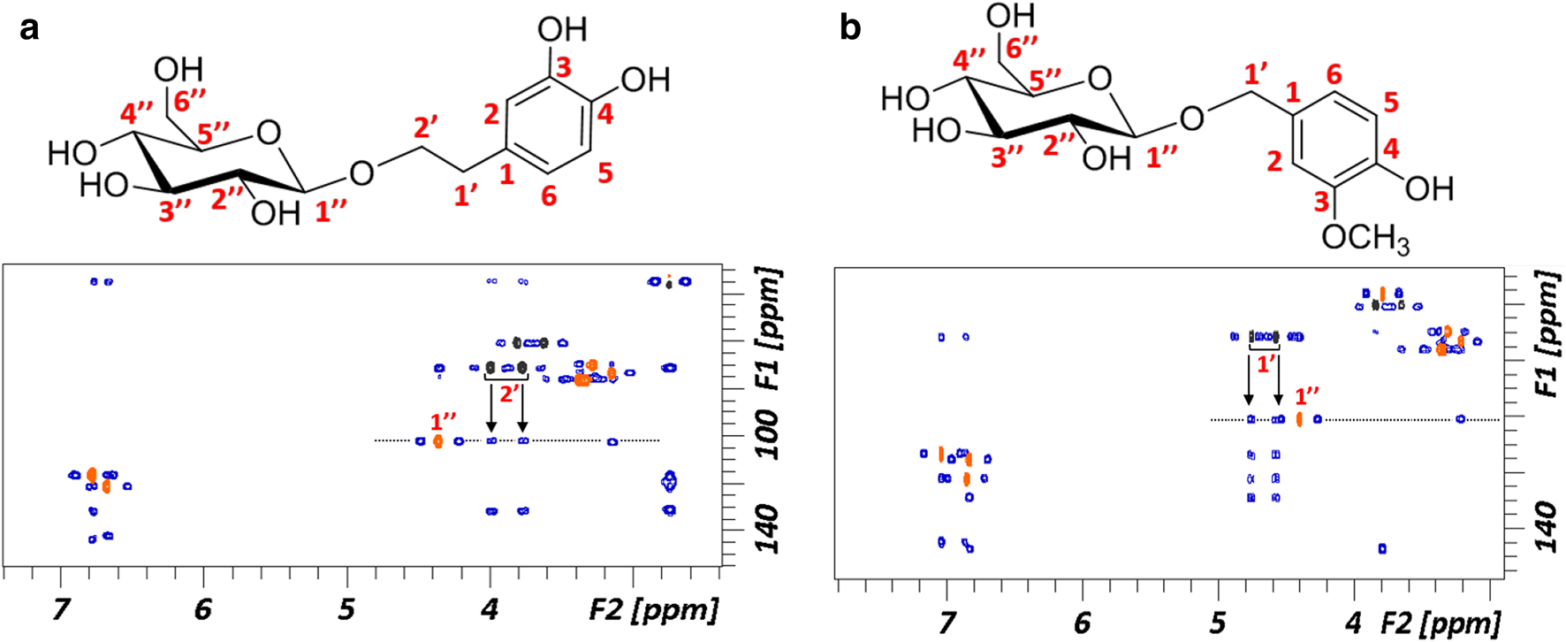
Superimposition of ^1^H-^13^C HSQC (orange/black) and HMBC (blue). Arrows represent the key cross peaks correlations corresponding to the connectivity between the anomeric position of glucose and the hydroxytyrosyl (**a**) and vanillyl (**b**) residues. Atoms are numbered in agreement with Table 4

^1^H and ^13^C-NMR experiments were carried out with the purified hydroxytyrosol and vanillyl glucosides produced in the reactions to assign their regiochemistry. The HMBC spectrum (Fig. 3) showed a correlation between the anomeric carbon (C1’’) and the carbon C2’ of the hydroxytyrosyl residue (see Table 4 with the 1H and 13C NMR chemical shifts). Moreover, the anomeric proton presents a coupling constant value of 8 Hz, pointing out the formation of the derivative through a β-linkage in accordance with the retaining β-glucosidase activity of the enzyme. Therefore, we can assure that the anomeric position is attached to the hydroxytyrosyl residue through the aliphatic chain. The same procedure was used to characterize the vanillyl glucoside, confirming that the anomeric position is connected to the vanillyl residue through the aliphatic chain (Fig. 3). This preference for the aliphatic chain versus the phenolic OH was also reported in a β-xylosidase of the same fungus, for the synthesis of hydroxytyrosol xyloside [21]. The complete NMR characterization procedure is explained in Data S2 and S3 (Additional file 1).

**Table 4:** Chemical shifts for hydroxytyrosol and vanillyl alcohol glucosides

	Hydroxytyrosol glucoside			Vanillyl glucoside	
	^1^H (ppm)	^13^C (ppm)		^1^H (ppm)	^13^C (ppm)
1″ Glu	4.36	102.2	1″ Glu	4.39	100.94
2″ Glu	3.15	73.25	2″ Glu	3.20	73.02
3″ Glu	3.35	76.38	3″ Glu	3.35	75.94
4″ Glu	3.27	69.65	4″ Glu	3.30	69.61
5″ Glu	3.35	76.38	5″ Glu	3.35	75.94
6″ Glu	3.62	60.60	6″ Glu	3.65	60.31
	3.81			3.82	
1 HT	–	128.97	1 Van	–	128.97
2 HT	6.77	113.29	2 Van	7.03	113.29
3 HT	–	147.46	3 Van	–	147.46
4 HT	–	144.86	4 Van	–	144.86
5 HT	6.77	115.37	5 Van	6.83	115.37
6 HT	6.67	121.36	6 Van	6.85	121.36
1′ HT	2.74	55.98	OCH_3_ Van	3.78	55.98
2′ HT	3.77	71.39	1′ Van	4.56	71.39
	3.99			4.76	

Final product yields are given in mM concentration

The fact that BGL-2 incorporates the glucosyl residue preferentially at the aliphatic hydroxyl could represent an advantage for the biological activity of these novel glucosides. The phenolic hydroxyl groups are the responsible for the antioxidative and free radical-scavenging activity of phenolic compounds, whose scavenging mechanism consists in donating the hydrogen atom of the phenolic –OH to free radicals, thus blocking their propagation during the oxidation process. The presence of a second and a third –OH in the phenolic ring could increase the antioxidant potential [38]. In this sense, it has been reported that the alkyl chain of caffeic acid and its derivatives, which are antioxidants with similar structure to the phenols evaluated in the current work, could have a role in stabilizing the radical formed during oxidation. However, its exact contribution remains uncertain [39]. Hence, the effect of glycosylation on bioactivity of the phenolic compounds should be further evaluated.

It is necessary to remark that both compounds can be found in some natural sources, as the vanillin glucoside in *Dendrotrophe frutescens* [40] or the hydroxytyrosol glucoside in *Prunus grayana* [41]. But the low yields obtained (0.032% and 0.067% of initial biomass according to both articles respectively), and the complex methodology, using a considerable amounts of organic solvents, that is required for obtaining both glycosides directly from plant extracts, makes transglycosylation an easier and efficient alternative in comparison.

### Antitumor potential of hydroxytyrosol and vanillin glucosides

It has been previously reported that hydroxytyrosol and vanillin and their derivatives can have an impact on different hallmarks of cancer [42, 43].

In this work, we analyzed if their glycosylation affects their antitumor activity, using the human breast cancer cell line MCF-7 and the human non-tumoral mammary epithelial cell line MCF-10A. We should remark that the action of vanillyl glucoside was compared with that of vanillin (instead of vanillyl alcohol) for its superior biological effects.

**Fig. 4:**
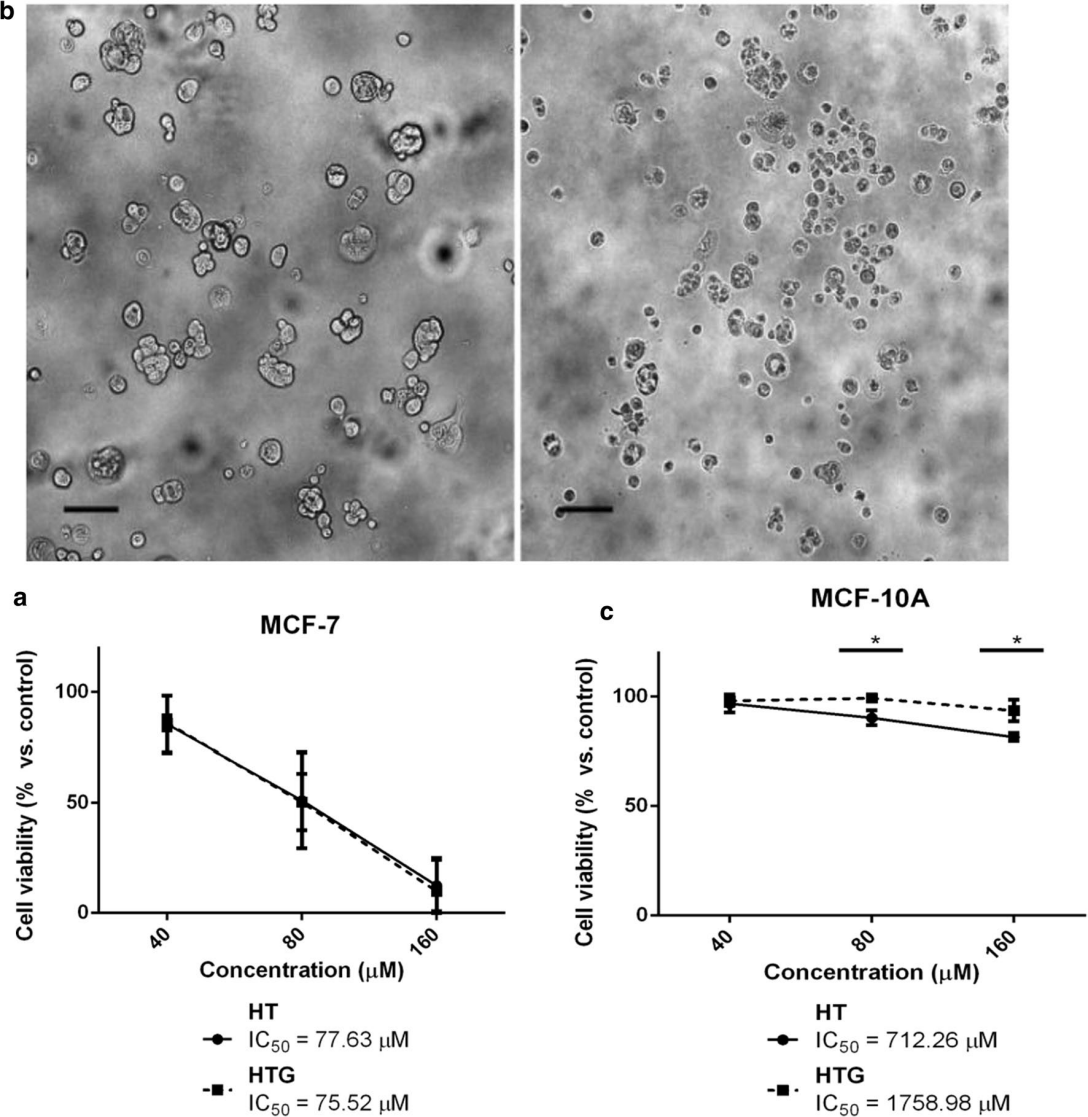
Effect of hydroxytyrosol and its glucoside on the viability of **a** MCF-7 and **c** MCF-10A cells after 24 h of compound addition. Cell viability was determined by crystal violet assay. Data represent mean ± SD of three independent experiments. *p < 0.05 vs. vehicle-treated cells. **b** Representative image of MCF-7 cell morphology after 24 h treatment with 160 µM hydroxytyrosol glucoside (right panel) or the corresponding vehicle (left panel). Scale bar, 100 µm

Results from crystal violet assays revealed that both, hydroxytyrosol and its glucoside remarkably reduced the viability of MCF-7 cells in a concentration-dependent manner (Fig. 4a). The concentrations inducing 50% decrease in cell viability (IC_50_) were very similar in both cases (Fig. 4a). This reduction was observed as soon as 24 h post compound addition, and it was accompanied by significant morphological changes in the cells (i.e. cell shrinkage, partial detachment and formation of apoptotic bodies) (Fig. 4b), suggesting apoptotic cell death. To investigate the safety profile of hydroxytyrosol glucoside and to compare it with the original phenol, we analyzed their effect on the viability of the non-transformed MCF10-A cell line. These cells were less sensitive to both, hydroxytyrosol and its glucoside, than their tumoral counterparts at every tested concentration (Fig. 4c).

The impact of this glucoside on the viability of non-tumoral cells was negligible, and therefore lower than that observed with the original compound (Fig. 4c). Overall, these results suggest that the glucoside has antitumor activity, with similar efficacy than free hydroxytyrosol but with a safer profile (i.e. less toxic on non-transformed cells).

It has been previously reported that hydroxytyrosol and hydroxytyrosol-rich olive leaves extracts decrease the viability of MCF-7 cells [44–46], as well as of other breast and non-breast cancer cell lines [42]. In most of these studies, this effect was attributed to the phenol’s capacity to inhibit cell proliferation and promote their apoptosis. Although some molecular mechanisms to explain this phenomenon have been proposed, the initial stress signals remain unknown. A number of recent reports concur that, although hydroxytyrosol is a compound well known for its antioxidant properties, under certain conditions, it can promote pro-oxidant effects and induce anti-proliferative and pro-apoptotic reactions in cancer cell lines through H_2_O_2_ generation [47, 48]. In addition, the same studies suggest that the *orto*-dihydroxy phenolic group present in this molecule, which is the main structural feature responsible for its free radical-scavenging activity, was also fundamental for the reported pro-oxidant effect.

As in the hydroxytyrosol glucoside, the phenolic OH is not involved in the transglycosylation reactions, there is no reason to think that the pro-oxidant properties may significantly differ from those of hydroxytyrosol. This would explain the almost identical decrease in the viability of MCF-7 cells observed upon addition of both compounds. Yet, further studies are needed to deepen our understanding on the molecular events underlying hydroxytyrosol/hydroxytyrosol glucoside-triggered cytotoxicity in the MCF-7 cancer cell line.

MCF-10A non-tumor breast epithelial cells, on the other hand, showed to be considerably more resistant to the deleterious effect of hydroxytyrosol and hydroxytyrosol glucoside than MCF-7 cells. It is interesting to point out that many human cancer cells present a highly oxidative state due to decreased antioxidant protective enzyme levels compared to their normal tissue counterparts. Therefore, cancer cells may be more sensitive to any generated reactive oxygen species (ROS) within the cells [45, 49]. The ability to trigger cell death specifically in cancer cells while not affecting non-cancerous cells is the basis for any potential antitumor compound and thus, hydroxytyrosol and its glucoside seems to be exceptional candidates to be so. In line with this notion, it is worth highlighting that while MCF10-A cells treated with hydroxytyrosol start displaying apoptotic features when challenged with concentrations up to 160 µM, hydroxytyrosol glucoside-treated ones barely show signs of cytotoxicity at all.

**Fig. 5:**
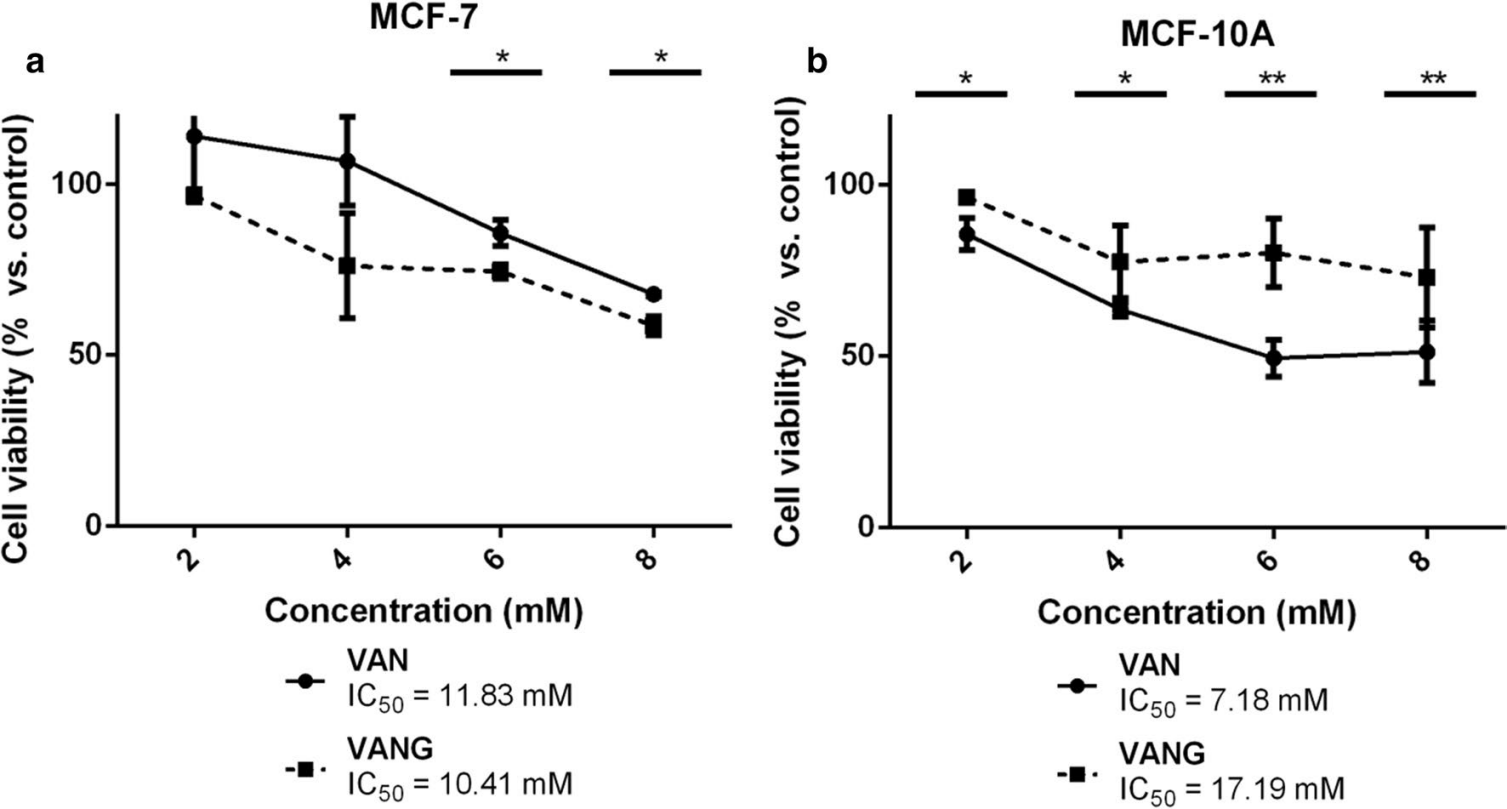
Effect of vanillin and vanillin glucoside on the viability of **a** MCF-7 and **b** MCF-10A cells after 72 h of compound addition. Cell viability was determined by crystal violet assay. Data represent mean ± SD of two (**a**) and three (**b**) independent experiments. *p < 0.05; **p < 0.01 vs. vehicle-treated cells

With respect to the potential antitumor action of vanillin and its glucoside, we monitored the effects of compound addition in MCF-7 and MCF-10A cells for several days. Figure 5a shows a concentration-dependent reduction in the viability of MCF-7 cancer cells in response to both compounds. The glycosylated derivative showed a slightly (but statistically significant) higher efficacy than the parental compound (Fig. 5a). It should be noted that these differences in cell viability between treated and untreated cells became visible only 72 h after compound exposure. In addition, no morphological differences could be detected by light microscopy as a consequence of compound treatment throughout the experiment (data not shown), suggesting that these compounds may be acting in MCF-7 cells as inhibitors of cell proliferation rather than as apoptotic stimuli.

In this case, and unlike the hydroxytyrosol pair, both vanillin and vanillyl glucoside significantly decreased the viability of non-tumoral MCF-10A cells (Fig. 5b). However, the glycosylated derivative showed a less toxic profile than vanillin.

Many lines of evidence support the potential beneficial effect of vanillin against human cancers [43]. Some authors have attributed its anti-invasive, anti-metastatic, anti-angiogenic and selectively cytotoxic actions to its ability to behave as a pro-oxidant instead of as an antioxidant [50], as we previously mentioned in the case of hydroxytyrosol. However, the mechanism through which vanillin modulates their selective anticancer effects has not been clearly delineated.

Our results showed no preferential cytotoxicity towards cancer cells after vanillin exposure. We hypothesize that the high concentrations used in this study (in the mM range) exerted toxic effects in both cell lines. As a matter of fact, aldehydes rarely accumulate in high concentrations in biological systems because of their high chemical reactivity. The natural vanillin biosynthesis pathway in the vanilla orchid, *Vanilla planifolia*, has an elegant solution to cope with the toxicity issue by glycosylation of vanillin to vanillin-β-D-glucoside [31]. Therefore, it is not surprising that vanillin glucoside displayed a weaker cytotoxic response on non-tumoral MCF-10A cells than vanillin.

In conclusion, glycosylation of vanillin generates a more effective anti-tumor compound that is less toxic to healthy cells than its non-glucosylated counterpart.

## Conclusions

In this work, the transglycosylation profile of three GH3 β-glucosidases (BGL-2, BGL-2T and BGL-3) was studied. The analysis showed that these enzymes may display transglucosylation in a variety of acceptors. BGL-2 was selected as model enzyme to corroborate the biotechnological potential of these enzymes because it displayed the highest transglucosylation yield. The results have shown that the glucosylated derivatives of hydroxytyrosol and vanillyl alcohol have more effective and/or safer profiles than hydroxytyrosol and vanillin when added to breast cancer cell cultures. The potential of this enzyme may lay the foundations for the design of new therapeutic tools for the management of cancer.

## Methods

### β-Glucosidase production and purification

BGL-2, BGL-2T, and BGL-3, from *T. amestolkiae* were heterologously expressed in *Pichia pastoris* and purified as previously reported [16, 17].

Enzymatic activity was determined spectrophotometrically at 410 nm by the release of *p*-nitrophenol (*p*NP) using *p*-nitrophenyl-β-D-glucopyranoside (*p*NPG) as substrate. One unit of β-glucosidase activity was defined as the release of 1 μmol of *p*NP per minute. Bovine serum albumin (BSA) was always added to every reaction (hydrolysis or transglycosylation), at final concentration of 0.1%, in order to get reproducible results and prevent loss of enzyme activity.

### Screening for potential transglycosylation acceptors

A library of 70 compounds, which can be seen in Table 1, was tested in a preliminary screening as potential transglycosylation acceptors by *T. amestolkiae* β-glucosidases. Stock solutions of these compounds were prepared in distilled water at 100 mM.

The assay was performed as described by Blanchard and Withers [24]. The first step consists on inhibiting the enzymes using 5 mM 2,4-dinitrophenyl 2-deoxy-2-fluoro-β-D-glucopyranoside, dissolved in 50 mM sodium phosphate (pH 6), at room temperature for 2 h. After inhibition, the sample was dialyzed against sodium phosphate pH 6, and an aliquot was added to each well of a 96-well plate. The final reaction mix was composed of each inactivated enzyme (in a dosage that would correspond to 2 U of β-glucosidase), 50 mM sodium phosphate buffer (pH 6), 0.1% BSA and 10 mM of each tested compound. Controls of non-inhibited enzyme and inhibited enzyme without acceptor were included in triplicate.

The plate was incubated for 1 h to allow the potential transglycosylation acceptors to reverse the enzymatic inhibition. After this time, *p*NPG was added to each well, and the change in absorbance at 410 nm was monitored for 30 min at 40 °C. The compounds that showed higher absorbance than the controls without acceptor were considered potential hits of transglycosylation.

### Analysis of the transglycosylation products by TLC and HPLC

After the aforementioned screening, the most interesting acceptors were assayed at higher scale. Transglycosylation standard reactions were carried out using 2.5 U/mL of each BGL in acetate buffer (pH 4.0), which is the optimum for BGL-2 variants and BGL-3 [16, 17].

100 mM cellobiose was used as donor and the different acceptors were tested in a concentration of 20 mM. The reactions were incubated for 1 h at 40 °C at 1200 rpm. Controls for each acceptor without enzyme were also carried out. The synthesis of glucosides was followed by thin layer chromatography (TLC) in order to confirm the positive hits. TLCs were carried out by using silica gel G/UV254 polyester sheets, (0.2 mm thickness and 40 × 80 mm plate size) provided by Macherey–Nagel, using ethyl acetate/methanol/water 10:2:1 (v/v) as running solution. Detection of compounds and glucosides was performed under 254 nm UV light.

After TLC screening, the transglycosylation products were also analyzed by High Performance Liquid chromatography (HPLC) on an Agilent 1200 series LC instrument equipped with a ZORBAX Eclipse XDB-C18 column (Agilent). The system was equilibrated in acetonitrile/H_2_O (9:91 v/v), both containing 0.1% acetic acid, with a flow of 2 mL/min, and the reaction products were separated isocratically for 8 min in the same buffer. Then, the mobile phase was changed to 95:5 acetonitrile/H_2_O, for washing the column for 3 min and the system was finally re-equilibrated to initial conditions for 4 min. The products’ peaks were detected by monitoring the absorbance at 270 nm. The peaks were quantified by referencing to a calibration curve of their phenolic precursors.

The purification of the selected glucosides was performed in the same HPLC equipped with a semi-preparative column (Mediterranea Sea 18 TR-010006, Teknokroma). The purification conditions were the same reported for HPLC analysis. After collection, the products were lyophilized and stored at − 20 °C.

### Analysis of the reaction products by mass spectrometry

The reaction mixtures were analyzed by conventional mass spectrometry, performed on a HCT Ultra ion trap. The samples were analyzed by electrospray ionization-mass spectrometry (ESI–MS) with methanol as ionizing phase in the positive reflector mode, and data were processed with the Masshunter Data Acquisition B.05.01 and Masshunter Qualitative Analysis B.07.00 software (Agilent Technologies).

### Optimization of transglycosylation catalyzed by BGL-2 by response surface methodology

The reaction conditions for the production of hydroxytyrosol and vanillin glucosides were optimized by a response surface methodology approach. Design-Expert^®^ software version 10.0.1.0 (Stat-Ease Inc. MN, USA) was used for generating a Box–Behnken design matrix and for data analysis. The parameters selected for building the model for glucoside production were the concentrations of the donor (cellobiose) and the selected acceptors (hydroxytyrosol and vanillyl alcohol), enzyme dosage, reaction time and temperature. The reactions were carried out at pH 4, the optimum for the enzyme [17]. After performing the required reactions, the software generates a polynomial quadratic equation from the obtained data, which reflects the effect of the independent variables on the response, and the highest production and yield expected.

### Solubility of novel glycosides

A saturated solution of hydroxytyrosol, vanillyl alcohol, and its glucosylated counterparts, were prepared in water and incubated at room temperature, at 500 rpm, during 2 h. Then, the solution was centrifuged, filtered, and analyzed with the HPLC, using the same protocol as previously described in the paragraph “Analysis of the transglycosylation products by TLC and HPLC”.

### Nuclear magnetic resonance (NMR)

NMR was used to confirm the structure and regiochemistry of the glucosides of hydroxytyrosol and vanillyl alcohol synthesized by transglycosylation. Samples were prepared in 500 μL of deuterated water (D_2_O) for analysis. NMR spectra were acquired at 298 K, using a Bruker AVANCE 600 MHz spectrometer equipped with a cryogenic probe. For spectral assignment one dimensional 1D ^1^H-NMR spectra, ^1^H-^13^C HSQC and HMBC experiments were acquired using corresponding pulse sequences included in TOPSIN acquisition software (Bruker).

### Cell lines and cultures

The human breast adenocarcinoma cell line MCF-7 was obtained from the American Type Culture Collection (ATCC) and maintained in Eagle’s Minimum Essential Medium (MEM) (Gibco by Thermo Fisher Scientific) supplemented with 10% fetal bovine serum (FBS) (Gibco), 10 μg/mL insulin (SAFC Biosciences by Sigma-Aldrich), 2 mM l-glutamin and 50 U/mL of a penicillin–streptomycin solution (Lonza). The human non-cancerous mammary epithelial cell line MCF-10A was obtained from the ATCC and cultured in Dulbecco’s Modified Eagle’s Medium (DMEM)/Ham’s F-12 (1 : 1) (Gibco) supplemented with 5% horse serum (HS) (Gibco), 10 μg/mL insulin (SAFC Biosciences), 0.5 μg/mL hydrocortisone (Sigma-Aldrich), 20 ng/mL epidermal growth factor (EGF) (Gibco), 10 μg/mL cholera toxin (Sigma-Aldrich) and 50 U/mL of a penicillin–streptomycin solution (Lonza). All cell lines were validated in the Genomics Core Facility at Alberto Sols Biomedical Research Institute (Madrid, Spain).

### Evaluation of cell viability by crystal violet staining

MCF-7 and MCF-10A cells were seeded in 96-well plates at a density of 4x10^3^ cells/well and allowed to attach to the plastic surface for 24 h. The medium was then replaced with fresh medium supplemented with hydroxytyrosol or hydroxytyrosol glucoside (40, 80 and 160 µM) and incubated for 24 h, or with 2, 4, 6 and 8 mM vanillin or vanillin glucoside for 72 h. Distilled water was used in both cases as vehicle. Following treatment, cells were incubated with 0.1% crystal violet (Panreac, Barcelona, Spain) for 20 min in agitation. The plate was then gently washed with tap water and the crystals were resuspended in methanol. Cell viability was determined by reading absorbance at 570 nm with a microtiter plate reader (Rayto Life and Analytical Sciences Co., Ltd., Shenzhen, China) and expressed as percentage versus vehicle-treated cells, set at 100%.

## Additional file

**Additional file 1:** Data S1. Equations for maximum production and maximum conversion for hydroxytyrosol and vanillyl glucosides. **Data S2.** RMN study of hydroxytyrosol glucoside. **Data S3.** RMN study of vanillin glucoside. 

## Data Availability

*Talaromyces amestolkiae* whole genome shotgun project is deposited at DDBJ/ENA/GenBank under the Accession Number MIKG00000000. BGL-2 sequence is deposited in GenBank under the Accession Number KM393203. BGL-3 sequence is deposited in GenBank under the Accession Number KM393202.1.
