# Supplementary material for: Transglycosylation products generated by Talaromyces amestolkiae GH3 β-glucosidases: effect of hydroxytyrosol, vanillin and its glucosides on breast cancer cells
Source: Microb Cell Fact. 2019 May 31;18:97. doi: 10.1186/s12934-019-1147-4 (PMC6544938; doi:10.1186/s12934-019-1147-4)
Supplement: Supplementary file 1 — Additional file 1: Data S1. Equations for maximum production and maximum conversion for hydroxytyrosol and vanillyl glucosides. Data S2. RMN study of hydroxytyrosol glucoside. Data S3. RMN study of vanillin glucoside. [file 12934_2019_1147_MOESM1_ESM.docx]

**TITLE**

Synthesis of glucosides by transglycosylation using GH3 β-glucosidases from *Talaromyces amestolkiae*: Hydroxytyrosol and vanillin glucosides as potential anticancer agents.

**Authors**: Juan Antonio Méndez-Líter^1^, Isabel Tundidor^2^, Manuel Nieto-Domínguez^1^, Beatriz Fernández de Toro^3^, Andrés González Santana^4^, Laura Isabel de Eugenio^1^, Alicia Prieto^1^, Juan Luis Asensio^4^, Francisco Javier Cañada^3^, Cristina Sánchez^2^, María Jesús Martínez^1^.

**Information of authors**:

^1^Department of Microbial and Plant Biotechnology, Centro de Investigaciones Biológicas, CSIC, Ramiro de Maeztu 9, 28040 Madrid, Spain.

^2^ Department of Biochemistry and Molecular Biology, Complutense University, Madrid, Spain; Instituto de Investigación Hospital 12 de Octubre, Madrid, Spain.

^3^ Department of Chemical and Physical Biology, Centro de Investigaciones Biológicas, CSIC, Ramiro de Maeztu 9, 28040 Madrid, Spain.

^4^Glycochemistry and Molecular Recognition Group. Instituto de Química Orgánica General (IQOG-CSIC), Calle Juan de la Cierva, 3, 28006 Madrid, Spain.

Data S1. Equations for maximum production and maximum conversion for hydroxytyrosol and vanillyl glucosides.

Hydroxytyrosol glucoside highest production was adjusted to the following quadratic model equation: Production (g/L) = - 0.039701 + 0.028607 * [BGL-2] - 3.25588E-003 * [Cellobiose] -2.35913E-003 * [HT] + 6.25378E-003 * Temperature + 0.10520 * Time + 3.20163E-004 * [BGL-2] * [Cellobiose] + 9.05336E-004 * [BGL-2] * [HT] - 3.86338E-004 * [BGL-2] * Temperature - 5.00206E-003 * [BGL-2] * Time + 1.59294E-004 * [Cellobiose] * [HT] + 9.09881E-005 * [Cellobiose] * Temperature + 1.31978E-003 * [Cellobiose] * Time + 3.55835E-004 * [HT] * Temperature + 3.60724E-003 * [HT] * Time - 3.52302E-004 * Temperature * Time - 2.08988E-003 * [BGL-2]^2^ - 1.95100E-005 * [Cellobiose]^2^ - 6.83017E-004 * [HT]^2^ - 1.41925E-004 * Temperature^2^ - 0.050764 * Time^2^.

Hydroxytyrosol glucoside highest conversion was adjusted to the following 2FI model equation: % conversion = + 2.90297 + 0.13209 * [BGL-2] - 0.045489 * [Cellobiose] - 0.017253 * [HT] -0.014176 * Temperature + 0.83868 * Time + 3.02077E-003 * [BGL-2] * [Cellobiose] -2.97914E-003 * [BGL-2] * [HT] -4.06229E-003 * [BGL-2] * Temperature - 0.062198 * [BGL-2] * Time - 3.79991E-004 * [Cellobiose] * [HT] + 8.56505E-004 * [Cellobiose] * Temperature + 0.012421 * [Cellobiose] * Time + 5.57314E-004 * [HT] * Temperature - 0.032373 * [HT] * Time - 6.90422E-003 * Temperature * Time

Vanillyl glucoside highest production also was adjusted to a quadratic model equation: Production (g/L) = -0.57667 + 0.061395 * [BGL-2] - 5.42711E-003 * [Cellobiose] + 2.40629E-004 * [Van] + 0.035012 * Temperature +0.21383 * Time + 5.14079E-004 * [BGL-2] * [Cellobiose] + 1.26264E-003 * [BGL-2] * [Van] - 7.16460E-004 * [BGL-2] * Temperature - 9.82830E-003 * [BGL-2] * Time + 2.99428E-004 * [Cellobiose] * [Van] + 1.28273E-004 * [Cellobiose] * Temperature + 1.96047E-003 * [Cellobiose] * Time + 3.10440E-004 * [Van] * Temperature + 6.00413E-003 * [Van] * Time - 3.65169E-003 * Temperature * Time - 3.26899E-003 * [BGL-2]^2^ - 3.14314E-005 * [Cellobiose]^2^ - 9.34766E-004 * [Van]^2^ - 4.64102E-004* Temperature^2^ -0.037594 * Time^2^.

The maximum conversion rate for Vanillyl glucoside was determined by the following 2FI equation: % conversion = + 3.20209 + 0.22437 * [BGL-2] - 0.071574 * [Cellobiose] - 0.015999 * [Van] + 0.034461 * Temperature + 2.52053 * Time + 4.99532E-003 * [BGL-2] * [Cellobiose] - 5.90886E-003 * [BGL-2] * [Van] - 7.25258E-003 * [BGL-2] * Temperature -0.085817 * [BGL-2] * Time - 5.53919E-004 * [Cellobiose] * [Van] + 1.13552E-003 * [Cellobiose] * Temperature + 0.019881 * [Cellobiose] * Time + 4.27289E-004 * [Van] * Temperature - 0.033424 * [Van] * Time - 0.048144 * Temperature * Time

[HT]: Hydroxytyrosol concentration. [Van]: Vanillyl alcohol concentration.

Data S2. RMN study of hydroxytyrosol glucoside

We herein describe the assignment of the hydroxytyrosyl-glucose derivative studied by NMR.


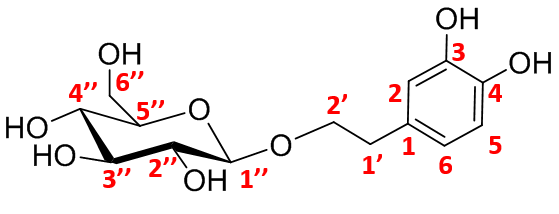


Structure of the hydroxytyrosyl-glucose derivative.

**NMR spectroscopy:** NMR experiments were acquired at 298 K, using a Bruker AVANCE 600 MHz spectrometer equipped with a cryogenic probe. 1D ^1^H NMR spectra, ^1^H-^13^C HSQC and HMBC experiments were acquired to assign all the NMR signals. For 1D ^1^H, ^1^H-^13^C HSQC and HMBC experiments, the zg, hsqcedetgp, and hmbcgpndqf sequences were employed.

HSQC correlates the chemical shifts of two types of nuclei (in this case ^1^H and ^13^C) that are directly bonded, while HMBC points out the correlation through two or more chemical bonds.


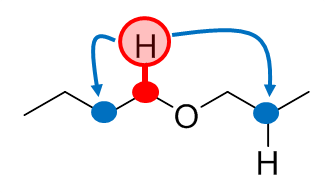


HSQC correlation (red) and HMBC correlation (blue).


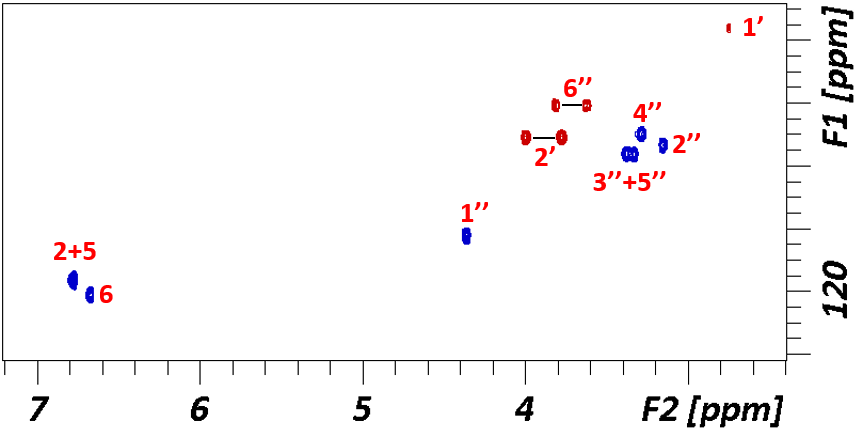
Labelled ^1^H-^13^C HSQC of the hydroxytyrosyl-glucose derivative.

We employ HMBC to observe the correlation between the anomeric carbon (**C1’’**) and the carbon **C2’** of the hydroxytyrosyl residue. Moreover, the anomeric proton presents a coupling constant value of 8 Hz, pointing out the formation of the derivative through a β-linkage.

Therefore, we can assure that the anomeric position is bonded to the hydroxytyrosyl residue through the aliphatic chain.


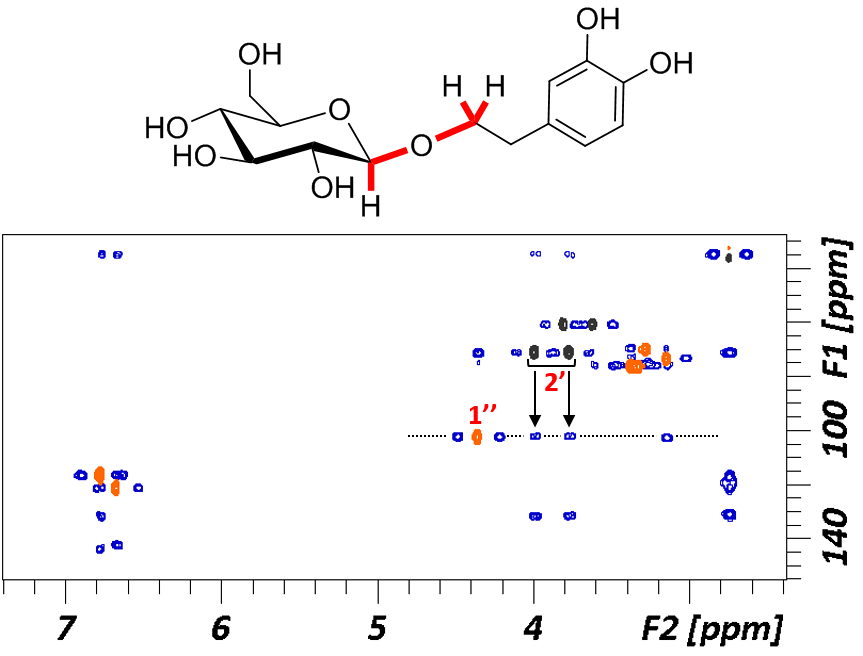


Superimposition of ^1^H-^13^C HSQC (orange/black) and HMBC (blue). Arrows represent the

key cross peaks for the characterization of the molecule, the correlation of the

anomeric position and the hydroxytyrosyl residue.

Data S3. RMN study of vanillin glucoside

We herein describe the assignment of the vanillyl-glucose derivative studied by NMR.


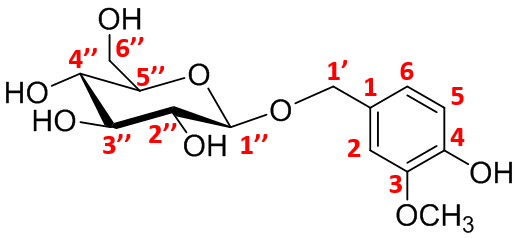


Structure of the vanillyl-glucose derivative.

**NMR spectroscopy:** NMR experiments were acquired at 298 K, using a Bruker AVANCE 600 MHz spectrometer equipped with a cryogenic probe. 1D ^1^H NMR spectra, ^1^H-^13^C HSQC and HMBC experiments were acquired to assign all the NMR signals. For 1D ^1^H, ^1^H-^13^C HSQC and HMBC experiments, the zg, hsqcedetgp, and hmbcgpndqf sequences were employed.


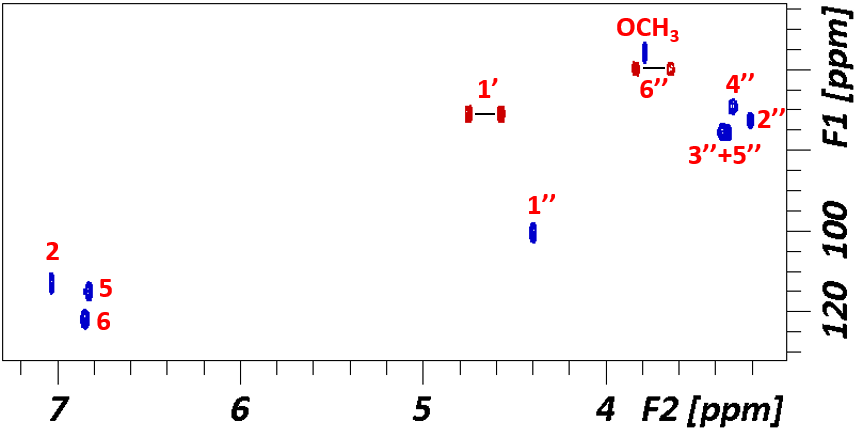
Labelled ^1^H-^13^C HSQC of the vanillyl-glucose derivative.

We employ HMBC to observe the correlation between the anomeric carbon (**C1’’**) and the carbon **C1’** of the vanillyl residue. Moreover, the anomeric proton presents a coupling constant value of 7.7 Hz, pointing out the formation of the derivative through a β-linkage.

Therefore, we can assure that the anomeric position is bonded to the vanillyl residue through the aliphatic chain.


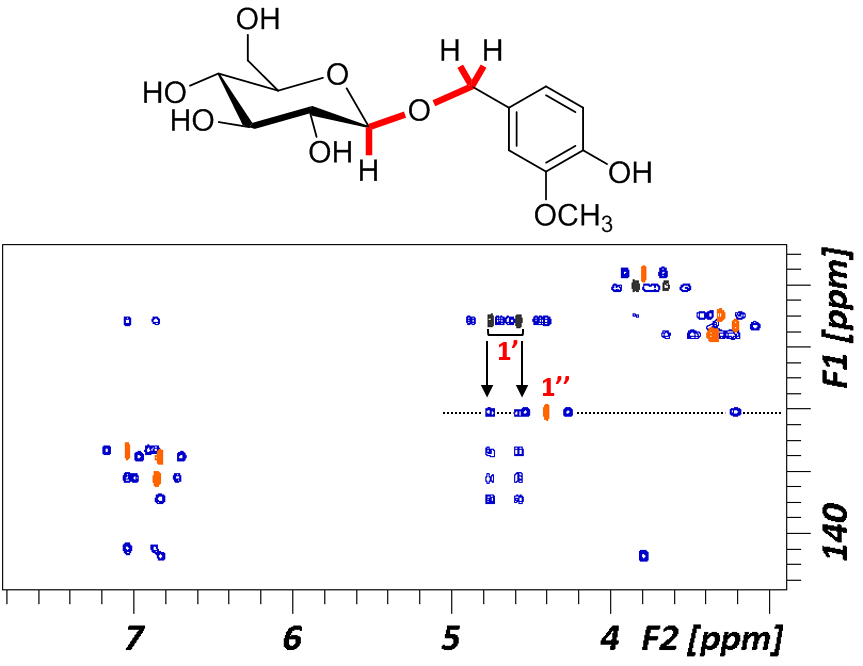


Superimposition of ^1^H-^13^C HSQC (orange/black) and HMBC (blue). Arrows represent the

key cross peaks for the characterization of the molecule, the correlation of the

anomeric position and the vanillyl residue.
